# Quackery and Christianity

**Published:** 1892-09-03

**Authors:** 


### QUACKERY AND CHRISTIANITY.

In every country of the world probably medical art in its earliest stages was surrounded by mysticism and quackery. The priestly mysticism which characterised the medical practice of some of the followers of Æsculapius in Greece was in its best days conducted with an honesty which only gradually degenerated into deception and trickery which human credulity, great as it is, could not put up with. The gross abuses which surrounded the temples of Æsculapius paved the way for their fall, which the introduction of Christianity completed.

